# Validation of caprine H11 and the Rosa26 platform for transgene integration via CRISPR-based system: investigations on stable transgene expression and genetic biosafety

**DOI:** 10.1007/s10142-025-01679-x

**Published:** 2025-08-29

**Authors:** Yaoguang Zhang, Fei Hao, Yuan Gao, Weiguo Song, Chang Su, Xudong Guo, Dongjun Liu

**Affiliations:** 1 State Key Laboratory of Reproductive Regulation and Breeding of Grassland Livestock, College of Life Sciences, Inner Mongolia University, Hohhot, 010070 China; 2 Alxa League Animal Quarantine Technology Service Center, Inner Mongolia, Alxa, 750300 China

**Keywords:** Genomic integration sites, *H11* locus, *Rosa26* locus, CRISPR/Cas9, Cashmere goat

## Abstract

CRISPR/Cas9 technology is an efficient tool for site-specific livestock gene editing. However, to minimize potential disruption of host genome function, exogenous genes should be integrated into well-characterized genomic loci, such as *H11* or *Rosa26*, which have been empirically validated for stable transgene expression. This study established a multi-dimensional assessment system to evaluate the biological applicability of the *H11* locus and the widely used *Rosa26* targeting platform as sites for targeted integration of exogenous genes in goats. Donor cells carrying the enhanced green fluorescent protein (*EGFP*) reporter gene at the *H11* and *Rosa26* loci were generated via CRISPR/Cas9-mediated homology-directed repair; this was followed by somatic cell nuclear transfer to produce transgenic cloned embryos and healthy offspring. Multi-dimensional analyses revealed the following. At the cellular level, there was stable and efficient *EGFP* expression at integration sites, with donor cells maintaining normal cell cycle progression, proliferation capacity, and apoptosis levels, and with no alterations in the transcriptional integrity of adjacent genes. At the embryonic level, there was sustained *EGFP* expression across pre-implantation embryonic stages, with developmental metrics statistically indistinguishable from wild-type embryos. Finally, at the individual level, cloned offspring exhibited growth phenotypes consistent with wild-type counterparts, and *EGFP* showed broad-spectrum expression in eight tissues. This study establishes the first CRISPR/Cas9-based crossscale (cellular–embryonic–individual) validation in goats, demonstrating that the *H11* and *Rosa26* loci support efficient and stable transgene integration in goats. These results provide a precise and predictable technical framework for livestock genetic improvement.

**Supplementary Information:**

The online version contains supplementary material available at 10.1007/s10142-025-01679-x.

## Introduction

The breakthroughs in gene editing technology this century are revolutionary owing to their precision and programmability, offering a novel paradigm for the genetic improvement of species. Among diverse genome-editing tools, the CRISPR/Cas9 system leverages sgRNA-guided targeting specificity and Cas protein nuclease activity, which induces targeted double-strand breaks (DSBs) (Jinek et al. 2012; Cong et al. 2013; Mali et al. 2013). These DSBs are subsequently repaired by cellular mechanisms: non-homologous end joining (NHEJ) leading to gene knockout, or homology-directed repair (HDR) when exogenous homologous templates are provided, enabling precise integration of exogenous genes (Lieber 2010; Sung and Klein 2006; Yang et al. 2020). This dual capability makes CRISPR/Cas9 uniquely suited for gene knock-in applications (Porteus and Baltimore 2003). Applications include livestock productivity enhancement, disease-resistant breeding, and disease model construction(Gim et al. 2023; Ledford 2023). Notably, complex editing tasks involving DNA fragment integration, such as gene knock-in, rely on CRISPR/Cas9-induced DSBs to initiate the cell’s endogenous HDR pathway for precise gene integration (Rouet et al. 1994).

Currently, exogenous gene integration faces technical bottlenecks, including endogenous gene disruption caused by random insertions (Eyquem et al. 2013). Site-specific integration strategies targeting"genomic safe harbors"have emerged as critical solutions to overcome transgenic limitations, ensuring stable, efficient transgene expression without compromising host genome integrity (Pagant et al. 2021). Ideal safe harbors require well-defined genomic localization, open chromatin structures, an absence of carcinogenic risks, and high predictability of integration events (Sadelain et al. 2011). Commonly targeted genomic sites identified across species include H11 (Hippenmeyer et al.^2010^) and the widely applied Rosa26 locus (Friedrich and Soriano 1991) in mice, and *CCR5* (C–C chemokine receptor type 5) (Lim et al. 2006) and *AAVS1* (adeno-associated virus integration site 1) (Smith et al. 2008)in humans. While *AAVS1* enables targeted integration, its limitations include: (1) susceptibility to adjacent regulatory interference (e.g., endogenous enhancers), and (2) functional disruption of the embedded tumor suppressor gene *PPP1R12C* (protein phosphatase 1 regulatory subunit 12 C) . Similarly, the *CCR5* locus contains cancer-associated genes that are susceptible to transgene dysregulation (Lombardo et al. 2011). Comparatively, the *H11* locus and *Rosa26*-targeting platform applied in model organisms such as mice demonstrate unique advantages. The *H11* site, located in an intergenic region of mouse chromosome 11, features an open chromatin structure enabling high-efficiency expression driven by exogenous promoters, with confirmed biosafety in artiodactyls, such as cattle and pigs (Hippenmeyer et al. 2010; Owen et al. 2021; Ma et al. 2022). The Rosa26 locus utilizes endogenous non-coding RNA promoters for ubiquitous transgene expression, exhibiting cross-species conservation from humans and cattle to sheep (Friedrich and Soriano 1991; Ma et al. 2022; Irion et al. 2007; Yuan et al. 2021; Wu et al. 2016).

Goats have substantial economic value as a globally important species in dairy, meat, and cashmere production. Recent CRISPR/Cas9 advancements in caprine genome engineering include: (1) dual knock-in/knock-out of rhBChE and *FGF5* via CRISPR/Cas9-nucleofection, achieving functional rhBChE secretion (Ellman assay) (Wang et al. 2023); (2) marker-free *Tβ4* knock-in at *CCR5* via CRISPR/Cas9, increasing cashmere yield by 74.5% without quality loss, with RNA-seq evidence of vascular modulation (Li et al. 2019); and (3) Complementary CRISPR/Cas9-mediated *FGF5* editing: (i) Embryonic disruption increasing hair follicle density/fiber length; (ii) *VEGF* co-integration with HDR enhancers, synergistically boosting cashmere yield/fiber length via PI3K-AKT/ECM (Wang et al. 2016; Hu et al. 2021). While recent studies have reported *Rosa26* locus editing in goats using transcription activator-like effector nucleases (Vats et al. 2021) and prime editing technology (Li et al. 2024), a systematic multi-dimensional evaluation comparing potential safe harbor loci (e.g., *H11* and *Rosa26*) in the context of CRISPR/Cas9-mediated gene editing and somatic cell nuclear transfer is still lacking. This gap hinders the advancement of precision genome editing in goats.

This study focused on cashmere goats, addressing key challenges, such as unstable transgene expression and low editing efficiency caused by random integration. We systematically investigated the functional conservation of *H11* and *Rosa26* loci in the caprine genome for the first time. Given the significant applications of transgenic goats in agricultural biotechnology and biomedicine, this study aimed to resolve the following: (1) the chromatin accessibility and genomic context of *H11/Rosa26* homologous loci in goats, (2) CRISPR-mediated site-specific integration systems, and (3) the transcriptomic impacts of exogenous gene integration. It is hoped that the conclusions of this study will fill technical gaL in caprine genome editing; enhance editing efficiency and safety; mitigate random integration risks; establish stable platforms for cashmere goat follicle development models, disease-resistant breeding, and bioreactor development; accelerate the translation of gene-editing technologies from basic research to industrial applications; and provide innovative solutions for genetic improvement and industry advancement.

## Materials and methods

### Prediction of *H11* locus and *Rosa26* targeting platform in goats based on cross-species genomic conservation

The genomic localization and sequence characteristics of the goat *H11* locus were determined by integrating genomic database searches with literature analyses. First, a cross-species conservation analysis was performed using the chromosomal coordinates of the adjacent genes *DRG1* and *EIF4ENIF1* to map the goat *H11* locus. Subsequently, multi-species (mouse, human, pig, sheep, and rabbit) *H11* locus sequences were aligned using the BLAST tool on the NCBI platform to evaluate evolutionary conservation and sequence homology. Finally, the core sequence and flanking regulatory regions of the goat *H11* locus were systematically predicted by combining the genomic localization data with cross-species conserved region analysis.

The goat *Rosa26* sequence and its conserved features were identified through multispecies homologous alignment and genomic database analyses. Based on literature searches, mouse (NC_000072.6) and sheep *Rosa26* sequences were retrieved. The Ensembl database was used for genomic localization, and the mouse 1 kb promoter and exon1 sequences were employed as probes to screen homologous regions in goats, successfully identifying goat *Rosa26* exon1. Further systematic alignment of the full sheep *Rosa26* sequence with the goat genome enabled prediction of the complete goat *Rosa26* sequence. To validate evolutionary conservation, a multi-sequence alignment was performed to analyze the homology of exon1 across goat, mouse, and sheep *Rosa26* genes, revealing species-specific sequence variations.

### Reverse transcription quantitative polymerase chain reaction (RT-qPCR)

Total RNA was extracted using RNAiso Plus (TaKaRa Bio, Kusatsu, Japan) and with a NanoDrop (Thermo Fisher Scientific, Waltham, MA, USA) spectrophotometer. Genomic DNA contamination was eliminated using gDNA Eraser (PrimeScript™ RT Reagent Kit) and verified via Melt curve analysis (single peak). cDNA was synthesized from 1 μg RNA using the PrimeScript RT Reagent Kit. RNA/cDNA were aliquoted and stored at –80 °C to prevent repeated freeze–thaw cycles. RT-qPCR was performed with TB Green® Premix Ex Taq™ II (TaKaRa Bio) on a CFX96 system using primers spanning exon-exon junctions (Supplementary Table S1). Data were analyzed via the 2^−ΔΔCt^ method with three biological replicates per group (*n* = 3) and three technical replicates per sample. Experimental groups included CRISPR/Cas9-edited *H11/Rosa26*-integrated cells/embryos/animals (*n* = 3), with wild-type controls (*n* = 3). This workflow adhered to the MIQE guidelines (Bustin et al. 2009). The primer sequences are listed in Table S1.

### Fibroblast cell culture

Goat fetal fibroblasts (GFFs) were isolated from 40-d-old embryos (Female) (Inner Mongolia Yiwei Cashmere Goat Co., Ltd., IACUC approval IMU-GOAT-2022–021) via tissue mincing and 0.25% trypsin digestion (30 min). Cells were cultured in DMEM/F12 supplemented with 10% FBS (Fetal Bovine Serum)and 1%(v/v) penicillin–streptomycin (100 × solution containing 10,000 U/mL penicillin and 10,000 μg/mL streptomycin) at 37 °C in a 5% CO_2_ incubator (Thermo Fisher Scientific) with real-time monitoring.

### Polymerase chain reaction (PCR)

Genomic DNA was extracted from GFFs using the Genomic DNA Kit (Tiangen Biotech, Beijing, China) and quantified with a NanoDrop spectrophotometer. PCR was performed with PrimeSTAR® HS DNA Polymerase (TaKaRa Bio) in a 25 μL reaction containing 12.5 μL 2 × PrimeSTAR Buffer, 0.2 mM dNTPs, 0.4 μM primers, 100 ng template DNA, and 0.625 U enzyme. Thermocycling: 98 °C for 3 min, 35 cycles of 98 °C (10 s), primer-specific annealing (15 s), and 72 °C (1 min/kb), followed by 72 °C for 5 min. Products were resolved on 1% agarose gels (1 × TAE buffer, Invitrogen) stained with GelRed™ (0.2 μg/mL, Biotium) and visualized using the Gel Doc XR + system (Bio-Rad) with the Image Lab 6.0 (v6.0.1; Bio-Rad Laboratories, Hercules, CA, USA). Images were captured in TIFF format without post-processing. The primer sequences are listed in Table S2.

### Construction of Cas9/gRNA co-expression vectors

The *Capra hircus* genome (NCBI: GCF_001704415.1) was used as a reference. Design tools included CRISPRdirect (DBCLS), CRISPOR, and CCTOP, with species-specific reference checks to ensure alignment with target sequences. Cas9/gRNA co-expression vectors were constructed using a commercial kit (Viewsolid Biotech, Beijing), with the oligos nucleotides shown in Table S3.

Plasmid purification and cloning validation:

Purification: Cas9/gRNA and *EGFP*-targeting plasmids were isolated via the PureYield Plasmid Midiprep System (Promega) and quantified using NanoDrop 2000 (Thermo Fisher Scientific).

Cloning verification: Sanger sequencing (BGI, China) using the vector-specific primer (sqprimer: TGAGCGTCGATTTTTGTGATGCTCGTCAG) confirmed the sequence of the inserted sgRNA fragment in the Cas9/gRNA co-expression vector. Sequences were aligned with reference loci (GenBank: *H11* and *Rosa26*) to confirm accuracy.

Selection markers: The *EGFP* gene served as a selection marker and transfection efficiency indicator. In *Escherichia coli* transformations, ampicillin resistance (Ampr) was used for selection.

### Precise evaluation of Cas9-sgRNA complex editing efficiency via TA cloning combined with Sanger sequencing

Cas9/gRNA co-expression vectors were transfected into 1 × 10^6^ GFFs using the NEPA GENE electroporator (Model: NEPA21 TYPE II, 250 V, 2.5 ms, Opti-MEM, Gibco). Transfection efficiency was assessed using fluorescence microscopy (Nikon TI-S) and quantified with ImageJ (v1.53a) based on fluorescence intensity of reporter gene-expressing cells. Editing activity assessment: Genomic DNA extraction: Forty-eight hours post-transfection, target regions (*H11* and *Rosa26* loci) were amplified using primers (Supplementary Table S2) based on the C. hircus genome (GCF_001704415.1). TA cloning: PCR products were denatured and renatured to form heteroduplexes, ligated into pMD19-T vectors (TaKaRa Bio), and transformed into DH5α competent cells. Sequencing & analysis: Randomly selected clones (BGI Group) were sequenced, with indel frequencies and base substitutions calculated via BLAST alignment to the reference genome.Plasmid purification: Cloned plasmids were purified using the PureYield Plasmid Midiprep System (Promega).

### Construction and validation of *H11/Rosa26* HDR vectors

Based on TA cloning results, sgRNAs with optimal cleavage efficiency were selected. Homology arms (~ 1,000 bp each) flanking the sgRNA target sites were amplified using PrimeSTAR® HS DNA Polymerase (TaKaRa Bio) at locus-specific annealing temperatures of 68 °C (*H11* left arm), 69.5 °C (*H11* right arm), 67 °C (*Rosa26* left arm), and 70 °C (*Rosa26* right arm) (primers sequences: Supplementary Table S2). Vector construction: Using the pEGFP-C1 plasmid (Fenghui Shengwu, Shanghai) as the backbone, the upstream homology arm was inserted via PciI/AseI restriction sites upstream of the *EGFP* coding sequence.The downstream homology arm was inserted via MluI restriction sites downstream of the *EGFP* coding sequence. Final vector: ~ 1,000 bp upstream homology arm + CMV promoter + *EGFP* + polyA + ~ 1,000 bp downstream homology arm.Validation:

Correct vector assembly was confirmed by restriction enzyme digestion using PciI, MluI, and AseI. Sequence integrity and reading frame accuracy were verified by Sanger sequencing.

### Cell recovery and cryopreservation

#### Cell recovery

Thaw cryopreservation tubes in 37 °C water bath, transfer cells to a centrifuge tube, and dilute cryoprotectant with 1:9 FBS/DMEM/F12 medium. Centrifuge (1200 rpm, 5 min), remove supernatant, resuspend in fresh 1:9 FBS/DMEM/F12 medium, and culture at 37 °C, 5% CO_2_, and saturated humidity.

#### Cell cryopreservation

Discard old medium, rinse with PBS, and digest cells with 0.25% trypsin (3 min, 37 °C). Terminate digestion with medium, centrifuge (1500 rpm, 5 min), and wash three times with PBS. Resuspend in 1:9 DMSO/FBS freezing solution, aliquot into cryovials, store at −80 °C for 24 h, then transfer to liquid nitrogen.

### Flow cytometry sorting and *EGFP*-integrated monoclonal cell screening

Plasmids (Cas9/gRNA and *EGFP*-targeting vector) were purified using the PureYield Plasmid Midi Extraction System (Promega). GFFs (obtained from a single 40-d-old female goat embryo, passage 3, karyotype 46, XX) were transfected with the co-expression vectors under optimized electroporation conditions and cultured in 6 μM RAD51 stimulating compound 1 (RS-1, Selleck Chemicals) to improve HDR efficiency. Forty-eight hours after transfection, *EGFP*-positive single cells were sorted into 96-well plates using a Sony MA900 cell sorter (488 nm blue laser for SSC/FSC detection, high purity mode: 100 μm nozzle, 30 psi pressure). After confirmation via PCR/Sanger sequencing, they were used as donor cells for SCNT.

### Off-target effect analysis

To ensure the safety of genome-edited goats, we predicted five potential off-target sites for sgRNAs targeting the H11 and Rosa26 loci using the CRISPOR algorithm (accessible at http://crispor.tefor.net/). PCR amplification followed by Sanger sequencing was performed to detect off-target effects at these sites. Predicted off-target loci and corresponding validation primers are detailed in Supplementary Tables S5, S6, and S7.

### SCNT-mediated production of gene-edited cloned goats

#### Oocyte collection and maturation

Ovaries from Alpine Cashmere Goats were collected from a commercial abattoir and transported in 30–35 °C saline (2–4 h). COCs (cumulus-oocyte complexes) were isolated by slicing ovarian follicles with a sterile scalpel into collection medium (pH 7.2–7.4, osmolality 280–300 mOsm/kg; recipe in Supplementary Table S8). On average, ~ 10 morphologically intact COCs (3 + layers of cumulus cells, uniform cytoplasm) were obtained per ovary pair. COCs were matured in vitro in maturation medium containing 1 μL/mL FSH (follicle-stimulating hormone; Ningbo Second Hormone Factory, China) and 1 μL/mL LH (luteinizing hormone; Ningbo Second Hormone Factory, China), with pH 7.2–7.4 and osmolality 280–300 mOsm/kg (full recipe in Supplementary Table S9). Maturation was performed at 38.5 °C under 5% CO_2_ for 24–30 h until first polar body extrusion (MII stage).

#### Reconstructed embryo construction

Donor cells (*H11/Rosa26*-*EGFP*-integrated fibroblasts) were cultured in DMEM/F12 + 20% FBS (VivaCell, China). MII oocytes were denuded with 0.1% hyaluronidase (Sigma, Germany) and enucleated in 0.025 mg/mL cytochalasin B (CCB, Sigma, Germany)-containing IVC medium using a micromani-pulator (removing ~ 1/3 cytoplasm + polar body). G0/G1-phase donor cells were injected into the perivitelline space to form reconstructed embryos.

#### Embryo activation and culture

Reconstructed embryos were activated with 5 μM Ionomycin (Sigma, Germany) in IVC medium (pH 7.2–7.4, osmolality 280–300 mOsm/kg; recipe in Supplementary Table S10) for 5 min, followed by 3.5 h in SOFaa + 2 mM 6-DMAP (Sigma, Germany) to inhibit polar body emission. Post-activation, embryos were cultured in embryo development medium (pH 7.2–7.4, osmolality 280–300 mOsm/kg; 38.5 °C, 5% CO_2_, 5% O_2_) until the 2–8-cell stage.

#### Embryo transfer and monitoring

Healthy 2–5-year-old does were synchronized with embryo developmental stages. Embryos were surgically transferred into the ampulla of the oviduct at the side of a corpus luteum or ovulation. Pregnancy was monitored via ultrasound (30, 60, and 90 d post-transfer). Postpartum, genomic integration of *H11*-*EGFP* and *Rosa26*-*EGFP* was verified via PCR and Sanger sequencing (Supplementary Table S2).

### Western blotting

Total protein was extracted using a mammalian protein extraction kit (CWBIO) and quantified via BCA assay (Thermo Fisher Scientific). For denaturation, 10 μg protein/lane was boiled in 5 × SDS Loading Buffer (Tris–HCl, SDS, glycerol, bromophenol blue, DTT) at 100 °C for 5–10 min. Proteins were separated on homemade 5% polyacrylamide gels (SDS-PAGE) and transferred to nitrocellulose membranes (0.45 μm pore size) using Tris–Glycine Transfer Buffer (Kangwei Century, China). Membranes were blocked with 5% skim milk and probed with primary antibodies against EGFP (1:3,000), and α-tubulin (1:5,000, internal loading control; all from Proteintech). After three 15 min TBST washes (Tris-buffered saline with 0.1% Tween 20), HRP-conjugated secondary antibodies (1:5,000) were applied. Signals were detected with ECL substrate (Thermo Fisher Scientific) and visualized via the Tanon 5200 imaging system (Tanon). β-actin normalized loading and transfer efficiency.

### Fluorescence microscopy of *EGFP* expression in nuclear transfer embryos

*EGFP* expression was monitored using an inverted fluorescence microscope (Nikon TI-S) with *EGFP*-specific filters.

### Tissue fluorescence detection

Cornual tissues were analyzed for *EGFP* intensity using a fluorescence imaging system with a 488 nm excitation light source (laser/LED). Owing to the constraints of rapid on-site imaging, physical scale markers were not included—this represents a limitation of the data presented. Postnatally cloned goats (transgenic/cloned) were euthanized at 1 year of age by intravenous injection of sodium pentobarbital (30 mg/kg), followed immediately by systemic perfusion with ice-cold PBS (4 °C) to clear blood. Key tissues including quadriceps femoris (muscle), left hepatic lobe (liver), and left ventricle (heart), were rapidly collected and processed via tissue fixation with 4% PFA (Solarbio, ready-to-use) at 4 °C for 24 h, followed by 1 × PBS rinsing. Histology procedure: Dehydration: Gradient ethanol (70% → 100%, 40 min–1 h/step); Clearing: Xylene (1:1 ethanol/xylene → pure xylene, 30 min/step). Paraffin embedding: 3 × molten paraffin infiltration (1 h/step), oriented embedding in molds, cooled at 4 °C. Sectioning: 5 μm sections cut via microtome, mounted on APES-coated slides, dried at 37 °C overnight after spreading at 42 °C. *EGFP* distribution was visualized under a Nikon TI-S fluorescence microscope, and fluorescence intensity (relative fluorescence units, RFU) was quantified using ImageJ and normalized against a wild-type negative control (a contemporaneous wild-type clone) (Animal Ethics Approval: IMU-GOAT-2022–021).

### Cell cycle analysis via PI staining and flow cytometry

For cell cycle analysis, three groups were tested: (1) wild-type (WT) cells (blank controls), (2) negative control (NC) cells transfected with empty vector plasmid, and (3) experimental groups with targeted integration at *H11* or *Rosa26* loci. Fixation: 1 × 10^6^ cells were fixed in 1 mL ice-cold 70% ethanol overnight at 4 °C. Centrifugation and washing: Cells were pelleted (1,500 rpm, 5 min), resuspended in 1 mL ice-cold PBS, and centrifuged again under identical conditions.Staining: Pellets were stained with PI/RNase A solution (25 μL PI stock + 20 μL RNase A in 1 mL staining buffer; 200–300 μL per sample) and incubated at 37 °C for 30 min in the dark.Flow cytometry: Analyzed using BD FACSCanto II (488 nm excitation), acquiring 30,000 events per sample. Gating was based on FSC/SSC to exclude debris/aggregates. Data Analysis: Performed using FlowJo v10.8.

### Apoptosis detection via Annexin-V/7-AAD staining

Apoptosis was assessed using Annexin-V-PE (5 μL) and 7-AAD (10 μL, 20 μg/mL) staining on 1 × 10^6^ GFFs. Cells were incubated with Annexin-V-PE in the dark for 5 min at 20–25 °C, followed by immediate 7-AAD addition before analysis on a BD FACSCanto II flow cytometer (488 nm excitation, PE 575/26 nm filter for Annexin-V-PE, 7-AAD 670 nm long pass filter). A minimum of 30,000 events per sample were acquired, with debris excluded via FSC/SSC gating and fluorescence data analyzed to distinguish early (Annexin-V-PE positive/7-AAD negative) and late/necrotic (Annexin-V-PE positive/7-AAD positive) apoptotic cells. AnnexinV-PE binds phos-phatidylserine on apoptotic membranes, while 7-AAD stains permeabilized cells. Controls included unstained cells, single-stained samples (AnnexinV-PE only or 7-AAD only), and EGFP-transfected cells without staining.

### CCK-8 assay for cell proliferation

Cells (5 × 10^3^/well) were seeded in 96-well plates and cultured for 24 to 120 h. Cell viability was assessed using 10% CCK-8 (diluted in regular cell culture medium; Yeasen Biotechnology) at a concentration of 110 µL/well. Absorbance at 450 nm was measured using a Thermo Scientific microplate reader (model: 300–1993), and background correction was performed using a blank control (culture medium + CCK-8 without cells). Data are presented as mean ± SD/SE (each condition was repeated three times), and statistical significance was determined by analysis of variance. Cell viability was calculated as follows:

$$\text{Cell viability}({\%})=\frac{\left[{\mathrm{A}}_{\left(\mathrm{treatment}\right)}-{\mathrm{A}}_{\left(\mathrm{black}\right)}\right]}{\left[{\mathrm{A}}_{\left(\mathrm{control}\right)}-{\mathrm{A}}_{\left(\mathrm{black}\right)}\right]}\times 100$$

### Developmental assessment of SCNT embryos

SCNT embryos were constructed by transferring donor cell nuclei (from WT or edited female goat fibroblasts; *n* = 3 independent cell lines per genotype) into enucleated oocytes. Oocyte enucleation was performed via microinjection in CCB-supplemented medium, and nuclear transfer was completed by electrofusion using an ECM 2001 system (BTX; 1.5 kV/cm, 30 μs, 2 pulses). Embryos were cultured in SOFaa-based IVCmedium at 38.5 °C under 5% CO_2_. Developmental progression (2-cell, 4-cell, 8-cell) was monitored every 12 h using a Nikon TI-S inverted microscope equipped with a 38.5 °C heating stage.

The cleavage rate was calculated as follows:

$$\text{Cleavage rate}\left({\%}\right)=\frac{\ge 2-\text{cell stage}}{\text{Total cultured embryos}}\times 100$$

Note: Embryonic development timelines were referenced against internal standards derived from the average of three WT goat SCNT cohorts owing to the absence of published benchmarks.

### Growth phenotype monitoring

Body weight, length, height, and chest circumference were measured in transgenic and WT goats at birth, 6 months, and 12 months using electronic scales (± 0.1 kg) and tape measures (± 0.5 cm). Data were analyzed descriptively.

### Ethics

All of these experiments were conducted in accordance with the National Research Council’s Guide for the Care and Use of Laboratory Animals. All protocols were approved by the Experimental Animal Care and Use Committee of Inner Mongolia University (protocol code IMU-GOAT-2022–021). All animal procedures were performed at Inner Mongolia Yiwei Cashmere Goat Co., Ltd.

### Statistical analysis

Intergroup comparisons of continuous variables were performed using independent samples t-tests (for two groups) or one-way analysis of variance (ANOVA) (for three or more groups). Data are presented as mean ± standard deviation (mean ± SD). Statistical significance was set at *α = 0.05*. Results are reported with t/F-values and exact p-values. Analyses were conducted using Prism 8.0 (GraphPad Software, San Diego, CA, USA).

## Results

### Cross-species conservation analysis and functional prediction modeling of caprine *H11* locus and *Rosa26* targeting platform

#### *H11* locus

**Fig. 1:**
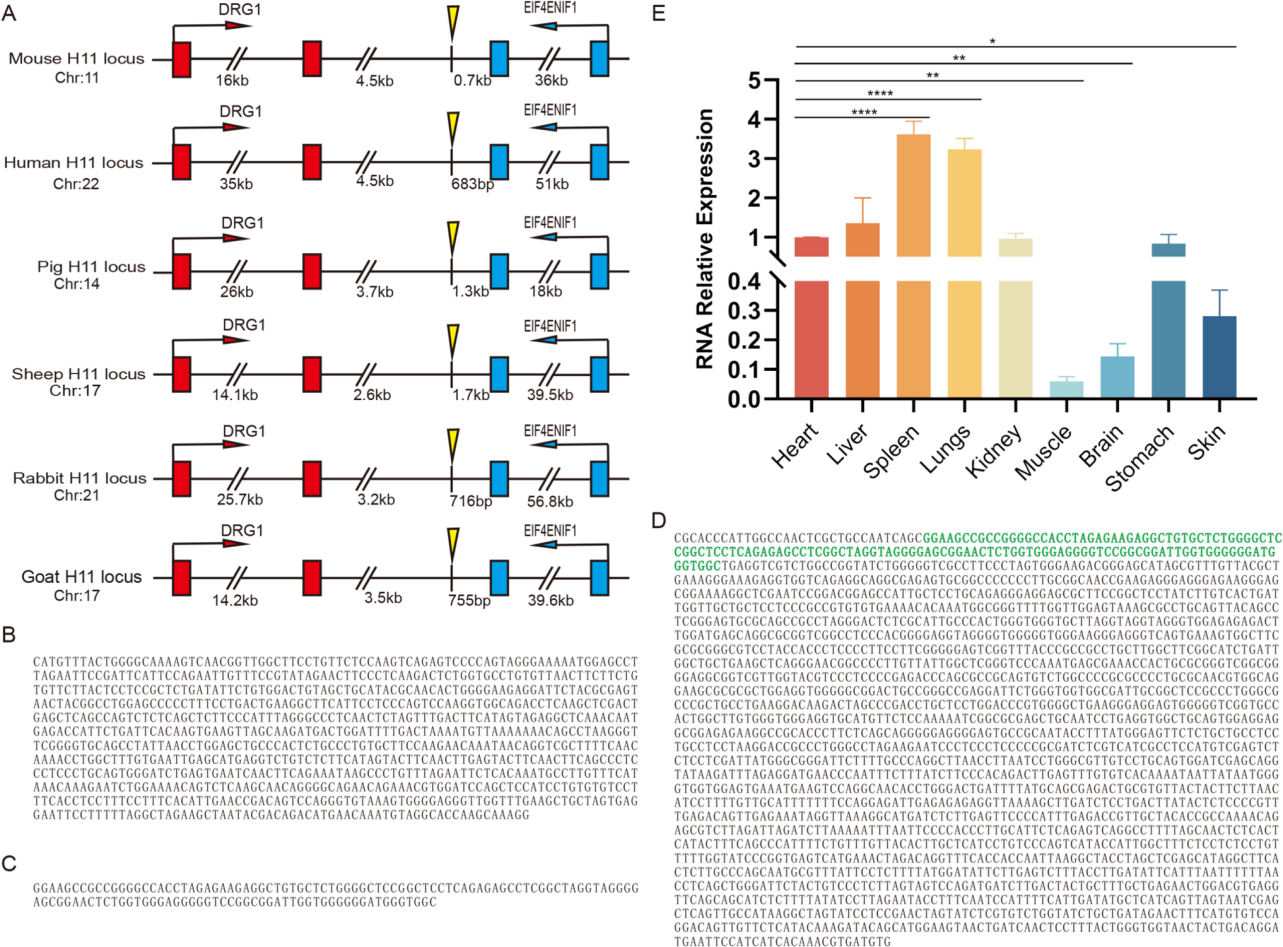
Genomic location and expression characteristics of *H11* and *Rosa26* loci in cashmere goats. (**A**) Schematic representation of the *H11* locus genomic context across species, generated with Adobe Illustrator; *DRG1* (red), *EIF4ENIF1* (blue), and predicted *H11* locus (yellow highlight). Chromosomal positions and flanking genes are labeled; (**B**) predicted sequence of goat *H11* locus; (**C**) first exon sequence of *Rosa26* gene; (**D**) The complete genomic sequence of the goat Rosa26 gene, the green part represents the first exon sequence; (**E**) relative expression levels of *Rosa26* transcripts in nine tissues (heart, liver, spleen, lung, kidney, muscle, brain, stomach, and skin). Data represent mean ± SD (*t*-test, *n* = 3). *p < 0.05, **p < 0.01, ***p < 0.001, **** *p* < 0.0001

By comparing genomic structural features across rodents (mouse), primates (human), artiodactyls (pig, sheep, goat), and lagomorphs (rabbit) using NCBI GenBank annotations, we observed conserved chromosomal adjacency of *DRG1* and *EIF4ENIF1* genes (≈5 kb spacing; Fig. S1A). Previous studies have identified the *H11* safe harbor locus in these species within this intergenic region (Owen et al. 2021; Ma et al. 2022). Based on cross-species conservation, we predicted the goat *H11* locus occupies this region and delineated its genomic coordinates and flanking sequences (Fig. S1B). Multiple sequence alignment (DNAman 6.0, Lynnon Biosoft, Canada; Lynnon 2022) revealed sequence similarities of 31.92% (mouse), 3.15% (human), 49.19% (pig), 38.64% (sheep), and 37.76% (rabbit) between the goat candidate region and *H11* homologs (Fig. S1C), indicating limited sequence conservation. Phylogenetic synteny analysis localized the core *H11* region in goats to a 3.1–4.1 kb interval downstream of *DRG1*, showing peak cross-species conservation (Fig. 1A). Genomic coordinates (*Capra_hircus* chromosome 17:69,574,165–69,575,165), validated via GenBank, overlap chromatin open-region features (Fig. 1B). Given *H11*’s compatibility with endogenous genes and its high transgene expression efficiency in other species, we propose this locus functions as a safe harbor in goats, enabling stable and efficient transgene integration without compromising genomic integrity.

#### *Rosa26* locus

Ensembl database comparisons revealed that the mouse (NC_000072.6) and sheep (Wu et al. 2016) *Rosa26* loci reside in a conserved intergenic region between *SETD5* and *THUMPD3* (Fig. S1D). Using the mouse *Rosa26* promoter and exon1 as probes, a 109 bp highly conserved region (92.66% homology) was identified on goat chromosome 22, overlapping 99 bp of the functional exon1 (Fig. S1E). NCBI BLAST alignments revealed a 590 bp homologous sequence (90% similarity) containing a 130 bp critical exon1 segment (Fig. S1F), enabling full prediction of the goat *Rosa26* exon1 (Fig. 1C). Alignment with sheep *Rosa26* sequences (98.78% similarity) identified a 2,295 bp region (chromosome 22:17,030,817–17,033,107; Fig. S1G). Multi-species alignment resolved the complete coding sequence of goat *Rosa26* (Fig. 1D), with exon1 similarities of 60.11% (goat vs. mouse) and 76.74% (goat vs. sheep) confirmed via DNAman 6.0 (Fig. S1H). RT-qPCR analysis of nine tissues (heart, liver, spleen, lung, kidney, muscle, brain, stomach, and skin) in WT cashmere goats revealed ubiquitous *Rosa26* transcript expression (Fig. 1E). Data were analyzed using independent samples t-test (*n* = 3 biological replicates), with significance levels indicated in the legend (*p < 0.05, **p < 0.01, ***p < 0.001).

### sgRNA screening and high-efficiency construction/validation of *H11/Rosa26* locus-specific homologous reintegration vectors

#### High-activity sgRNA screening and CRISPR/Cas9 system optimization

**Fig. 2:**
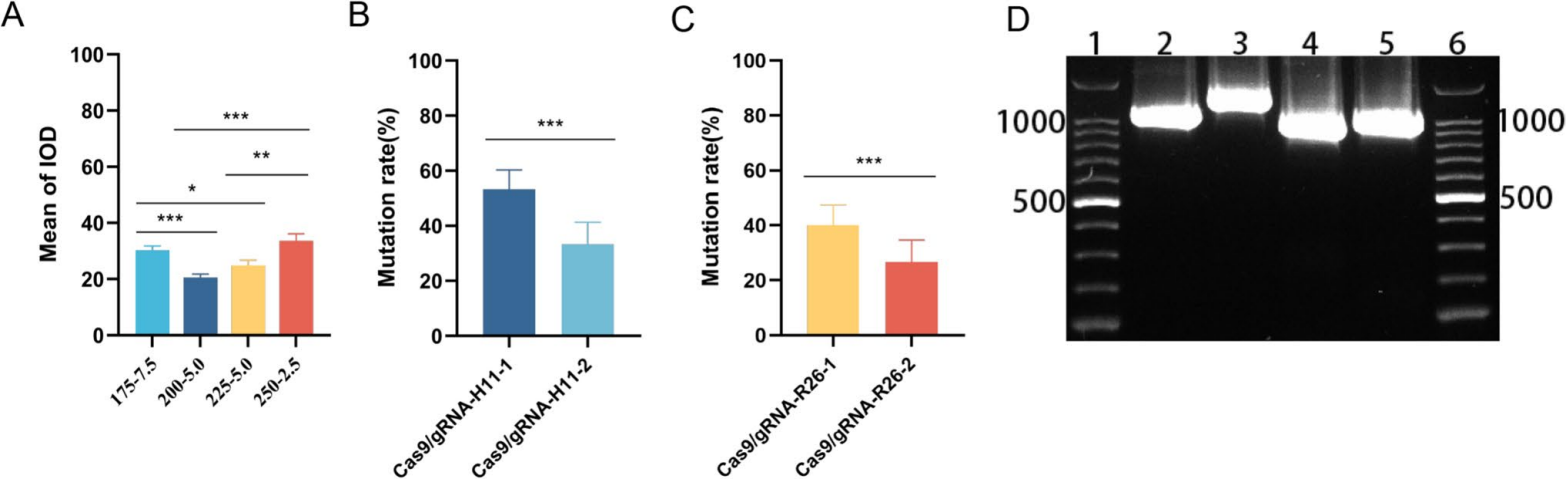
Design of the CRISPR/Cas9 gene editing system and validation of targeting vector construction (paired t-test, * *p* < 0.05; ** *p* < 0.01; *** *p* < 0.001). (**A**) Fluorescence intensity of plasmids under different transfection conditions. Data represent mean ± SD (*n* = 3). (**B**) Editing efficiency statistics of *H11* locus-targeting sgRNAs. Data represent mean ± SD (*n* = 3). (**C**) Editing efficiency statistics of *Rosa26* locus-targeting sgRNAs. Data represent mean ± SD (*n* = 3). (**D**) Agarose gel electrophoresis of homology arm PCR amplification products. Lane (PCR product): 1&6: DNA marker; 2: *H11* left homology arm; 3: *H11* right homology arm; 4: *Rosa26* left homology arm; 5: *Rosa26* right homology arm

Based on the identified goat *H11* and *Rosa26* locus sequences, two high-scoring sgRNAs (sgRNA-H11-1/−2 and sgRNA-R26-1/−2) were screened for each locus using bioinformatics tools (CRISPRdirect, CRISPOR, and CCTOP). Cas9/sgRNA co-expression vectors were constructed (Fig. S2A, B), and the electroporation parameters were optimized to 225 V/2.5 ms (Fig. 2A, S2E; Table S4). TA cloning analysis showed that the targeting efficiency of sgRNA-*H11*−1 at the *H11* locus was 53.33% (Fig. 2B), significantly higher than that of sgRNA-*H11*−2 (33.33%, *** *p* < 0.001 by paired t-test). At the *Rosa26* locus, the targeting efficiency of sgRNA-*R26*−1 was 40%, which was better than that of sgRNA-*R26*−2 (26.67%, *** *p* < 0.001 by paired t-test) (Fig. 2C). sgRNA-*H11*−1 and sgRNA *R26-*1 were selected for subsequent experiments.

#### Construction of *H11/Rosa26* locus-specific homologous reintegration vectors

Using the selected sgRNAs, *H11*-*EGFP*-donor and *R26*-*EGFP*-donor gene targeting vectors were assembled (Fig. S2C, D). PCR amplification confirmed the presence of left and right homology arms (*H11*-L/R arms and *R26*-L/R arms) (Fig. 2D). The homology arms were cloned into the pMD19-T vector and directionally inserted into the pEGFP-C1 backbone. The resulting vectors *H11*-*EGFP*-donor and *R26*-*EGFP*-donor were verified via gel electrophoresis.

### CRISPR/Cas9-mediated gene-edited cell line construction and high-efficiency generation of cloned goats

#### Establishment and validation of safe harbor-specific gene-targeted cell lines

**Fig. 3:**
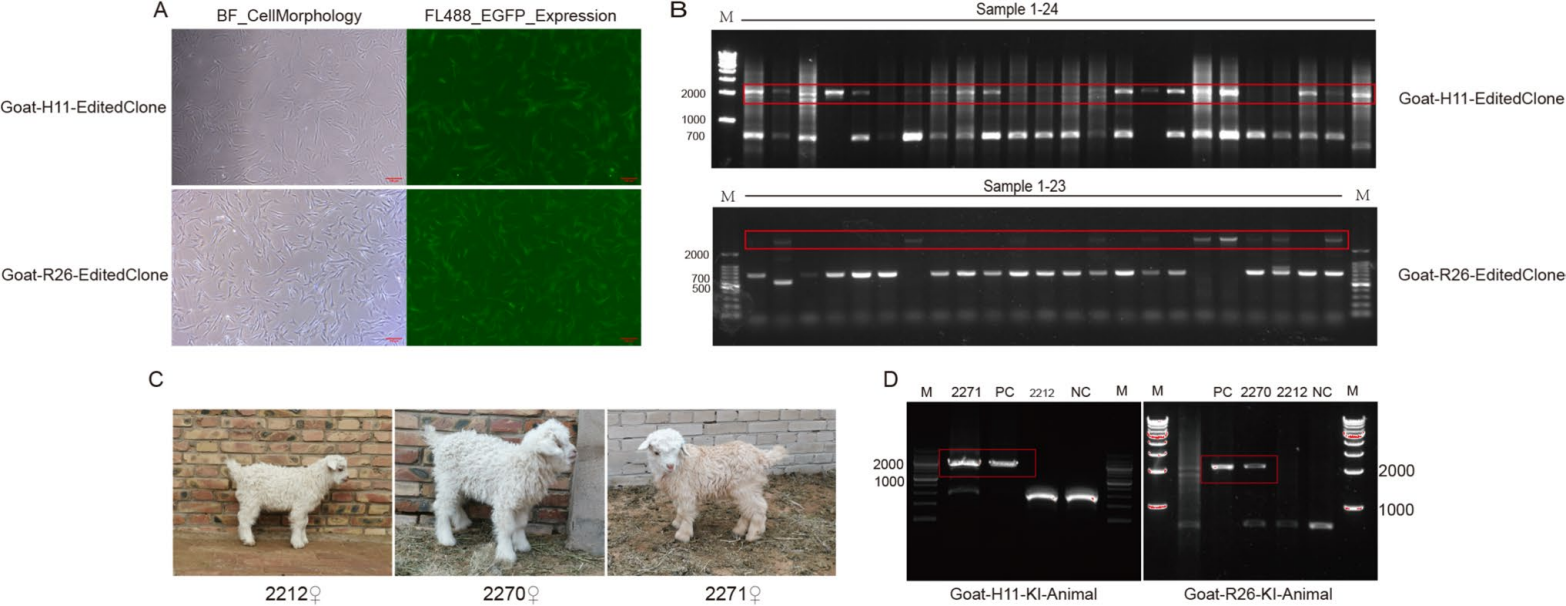
Screening of targeted integration cell lines and phenotypic/genotypic validation of cloned kids. (**A**) *H11*/*Rosa26* locus gene editing cells (1 month old) (Scale bar = 100 μm). (**B**) Agarose gel electrophoresis image validating targeted integration in the Goat-*H11*-EditedClone series and Goat-*R26*-EditedClone series. The NC is shown in Supplementary Fig. S6. (**C**) Phenotypic photographs of cloned kids post-birth (designated Goat-*R26*-KI-Animal 2270, Goat-*H11*-KI-Animal 2271, SCNT-WT-Animal 2212) (1-month-old femals). (**D**) Gene-edited cloned kids: Targeted integration genotyping via PCR -electrophoresis (peripheral blood samples)

Cotransfection of Cas9/sgRNA-*H11*−1 with the *H11*-*EGFP*-donor vector and of Cas9/sgRNA-*R26*−1 with the *R26*-*EGFP*-donor vector under identical conditions was followed via flow cytometry-based sorting and continuous culture, yielding 54 monoclonal cell lines. PCR and sequencing validation revealed that 19 of 28 H11-targeted clones (67.86%) achieved precise *EGFP* integration (designated as Goat-*H11*-EditedClone 01 to Goat-*H11*-EditedClone 19), whereas 12 of 26 *Rosa26*-targeted clones (46.15%) showed successful integration (designated as Goat-*R26*-EditedClone 01 to Goat-*R26*-EditedClone 12). The validated positive clones were cryopreserved for further use (Fig. 3A, B). The PCR products of each potential off-target site were sequenced and compared with the WT PCR products. The results are shown in Figs. S3 and S4. The sequence homology was found to be 100%, with no off-target phenomenon occuring at the potential off-target sites of *H11* and *Rosa26*. The above results show that sgRNA1 is not only highly efficient, but also has a low off-target rate, and is a good choice for site-specific integration of exogenous genes.

#### SCNT and molecular characterization of safe harbor-edited cloned goats

Donor cells with *EGFP* integrated into the *H11* or *Rosa26* loci were transplanted into enucleated oocytes. From 1,722 mature oocytes, 1,028 reconstructed embryos were generated (59.7% reconstruction efficiency), of which 498 successfully entered the cleavage stage. Among embryos in the cleavage stage, 44 exhibited no site-specific modifications (WT donor cells), while 249 and 205 embryos showed successful modifications at the *H11* and *Rosa26* loci, respectively (Table S11). A total of 359 embryos were transferred into recipient does via 107 transplantation procedures, resulting in three healthy full-term cloned kids (0.8% efficiency), designated #2212 female, #2270 female, and #2271 female (Fig. 3C). The 359 embryos transferred were categorized as follows (Table 1): Molecular analyses confirmed that kid #2270 showed the expected 2,056 bp band at the *Rosa26* locus and was named Goat-*R26*-KI-Animal 2270; kid #2271 exhibited the expected 2,157 bp band at the *H11* locus and was named Goat-*H11*-KI-Animal 2271; kid #2212 lacked the target bands and was named SCNT-WT-Animal 2212 (Fig. 3D). Given the limited sample size, the physiological data are preliminary observations.

**Table 1:** Embryonic development and transplantation outcomes

Embryo type	Total	2-cell	4-cell	8-cell	Uncleaved
WT	14	6	6	2	0
*H11* locus editing embryos	177	69	63	25	20
*Rosa26* locus editing embryos	168	33	80	37	18

### Validation of high-efficiency *EGFP* expression across developmental stages mediated by *H11/Rosa26* loci and tissue-specific distribution analysis

#### Quantitative analysis of *EGFP* expression at the cellular level

WT: Untransfected and unedited primary goat fibroblasts. NC: Cells transfected with the empty vector backbone plasmid. Positive Control (PC): Cells transiently transfected with the pEGFP-C1 plasmid for 48 h.

**Fig. 4:**
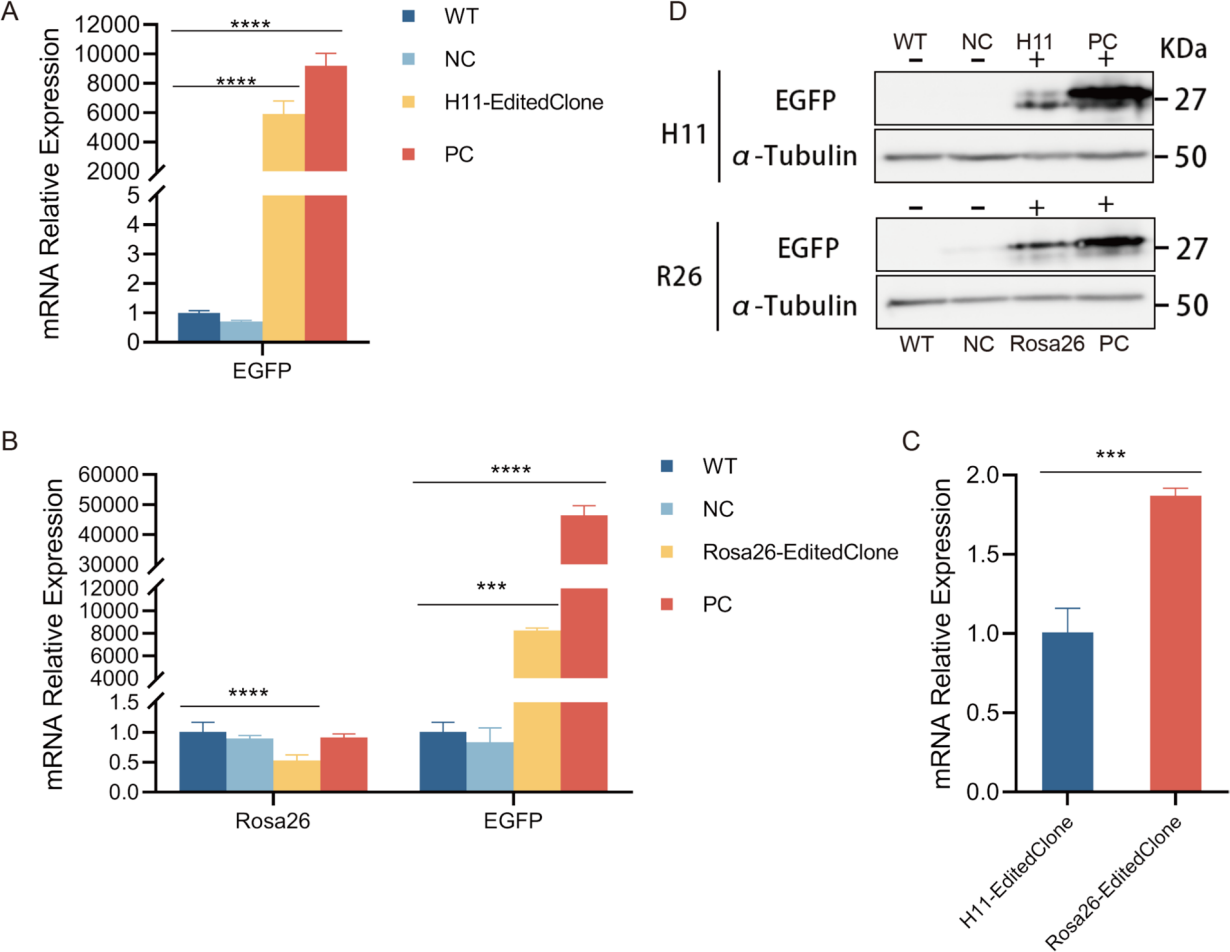
Cross-level expression analysis of *EGFP* mediated by *H11* and *Rosa26* loci at the cellular level. (**A**) Relative mRNA expression levels of *EGFP* at the *H11* locus. Data represent mean ± SD (*n* = 3). Statistical significance: *** *p* < 0.001;**** *p* < 0.0001. (**B**) Comparative mRNA expression profiles of the endogenous *Rosa26* gene and *EGFP* at the *Rosa26* locus. Data represent mean ± SD (*n* = 3). Statistical significance: *** *p* < 0.001;**** *p* < 0.0001. (**C**) Cross-comparison of *EGFP* mRNA expression levels at *H11* and *Rosa26* loci. Data represent mean ± SD (*n* = 3). Statistical significance: *** *p* < 0.001. (**D**) EGFP protein expression validation by Western blot. Upper panel*: EGFP* expression from the *H11* locus; Lower panel: *EGFP* expression from the *Rosa26* locus (*n* = 3). Original data are provided in the compressed file: WB_RAW_Annotated_EGFP-H11-Rosa26_20250701.zip

RT-qPCR analysis revealed that *EGFP* expression in the *H11*-targeted group (Goat-*H11*-EditedClone series) was 9,200-fold higher than that in the WT control (*p* < 0.0001 by one-way ANOVA with Tukey's post-hoc test), significantly exceeding that in the NC (0.70-fold, *p* > 0.05) and PC (5,917-fold, *p* < 0.0001). For the *Rosa26*-targeted group (Goat-*R26*-EditedClone series), *EGFP* expression increased 46,416-fold compared with that of the WT(*p* < 0.001 by one-way ANOVA with Tukey's post-hoc test), far surpassing that of NC (0.84-fold, *p* > 0.05) and PC (8,270-fold, *p* < 0.001), confirming that both loci support high-efficiency transgene expression (Fig. 4A, B).

Crucially, in gene-edited cell lines (Goat-*R26*-EditedClone series), endogenous *Rosa26* transcript levels exhibited a marked reduction (47%, Student's t-test, *p* < 0.01) compared to WT controls, confirming that transgene integration disrupted native gene expression. This observation provides direct molecular evidence for precise on-target integration at the *Rosa26* locus. By integrating genomic conservation and expression profiles, we confirmed that the goat *Rosa26* locus is a functional non-coding RNA platform suitable for transgene integration, providing a theoretical foundation for transgenic applications (Fig. 4B). Comparative analysis showed that the Goat-*R26*-EditedClone series exhibited significantly higher *EGFP* expression (at the RNA level) than the Goat-*H11*-EditedClone series (Fig. 4C; Student's t-test, *p* < 0.001; primer sequences are listed in Table S1).

Western blotting detected *EGFP*-specific bands in *H11*/*Rosa26*-targeted groups and PC, but not in WT or NC. Grayscale analysis indicated 1.25-fold higher *EGFP* protein expression in the Goat-*R26*-EditedClone series than in the Goat-*H11*-EditedClone series (Fig. 4D; Student's t-test, *p* < 0.05), consistent with the RNA level. These results demonstrated robust and stable *EGFP* expression at both the H11 and *Rosa26* loci. Stable integration was confirmed in all edited clones used for analysis by genomic PCR amplification spanning the integration junctions followed by Sanger sequencing, distinguishing them from transiently expressing PC cells. Cells were cultured for approximately 15–20 d post-thawing to obtain monoclonal populations. Prior to transfection or analysis, cells were typically passaged once to ensure optimal recovery and metabolic state.

#### *EGFP* expression validation across embryonic developmental stages

**Fig. 5:**
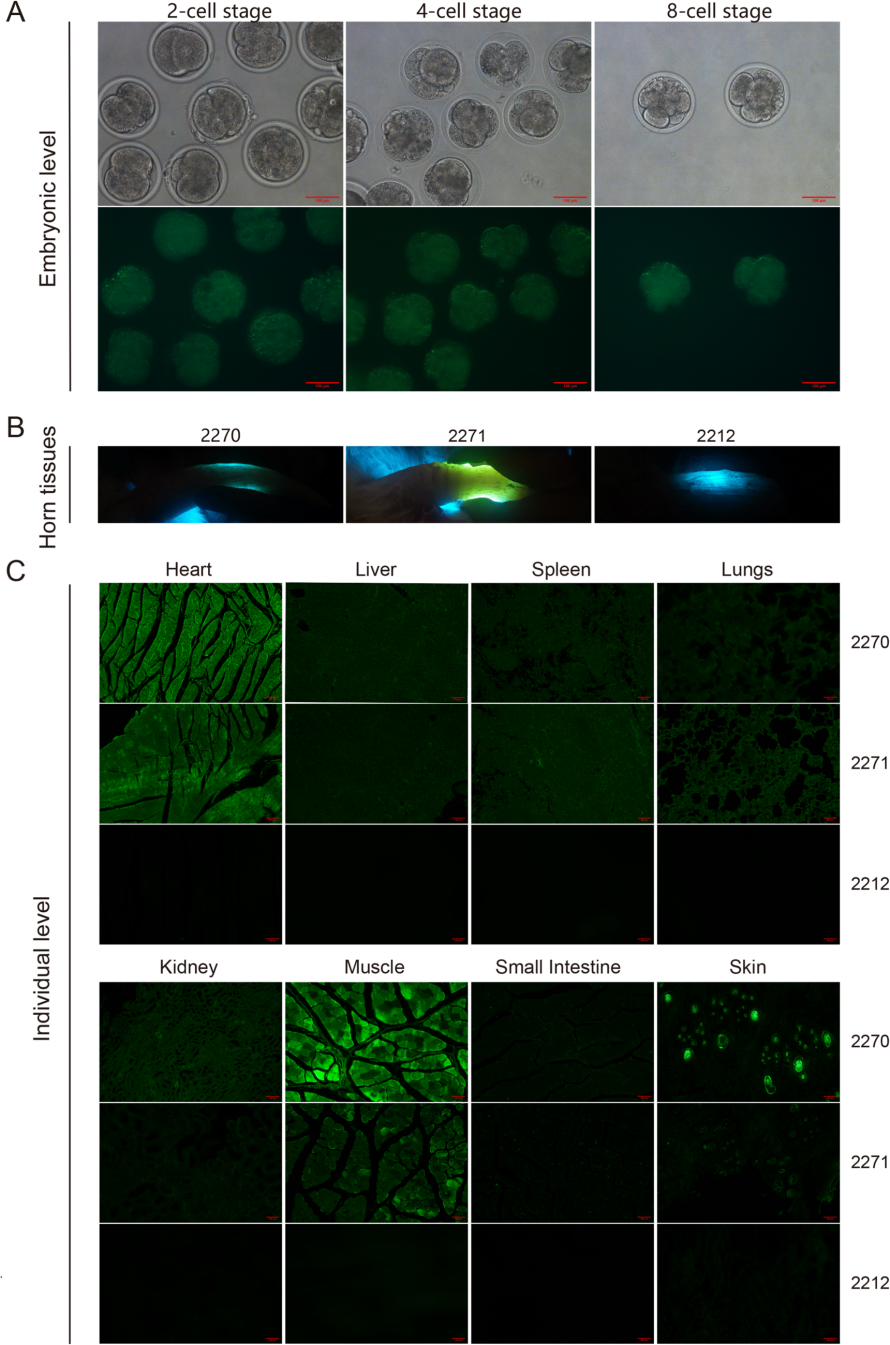
Cross-level expression analysis of *EGFP* mediated by *H11* and *Rosa26* loci. (**A**) Fluorescence expression of *EGFP* in nuclear transfer embryos (scale bar = 100 μm). (**B**) Field-based horn fluorescence screening in cloned kids.(lack of physical ruler). (**C**) Multi-tissue *EGFP* expression distribution (scale bar = 100 μm)

Prior to embryo transfer, inverted fluorescence microscopy revealed strong green fluorescence signals in both the Goat-*H11*-EditedClone series- and Goat-*R26*-EditedClone series-derived reconstructed embryos, confirming efficient *EGFP* expression during early embryogenesis (Fig. 5A).

#### Tissue-specific *EGFP* expression profiling in cloned individuals

Portable fluorescence imaging detected distinct *EGFP* signals in the horn tissues of Goat-*H11*-KI-Animal 2271 and Goat-*R26*-KI-Animal 2270 (Fig. 5B; note: corresponding brightfield images were not acquired). Fluorescent section analysis confirmed *EGFP* positivity in the heart, liver, spleen, lung, kidney, muscle, small intestine, and skin (Fig. 5C). Quantification of fluorescence intensity using ImageJ revealed significantly higher levels in Goat-*R26*-KI-Animal 2270 compared with those in Goat-*H11*-KI-Animal 2271 in specific tissues (Fig. S5A). Notably, Goat-*R26*-KI-Animal 2270 exhibited significantly higher fluorescence intensity in the heart (mean 26.48 vs. 23.45) and skin (26.16 vs. 12.52) than Goat-*H11*-KI-Animal 2271 (*p* < 0.05 by independent samples t-test for each tissue), with heart and skin signals 2-3-fold higher than those in other tissues (e.g., liver: 8.68; spleen: 6.23), indicating tissue-specific expression bias. In Goat-*R26*-KI-Animal 2270, the small intestine (19.92) and muscles (10.01) also showed relatively high expression levels. In contrast, Goat-*H11*-KI-Animal 2271 exhibited a broad-spectrum expression trend in specific tissues (e.g., heart, liver), but displayed organ-specific expression differences (Fig. S5B).

### Multi-dimensional safety assessment of transgene integration: Full-cycle analysis from cellular function to cloned individual development

#### Systematic evaluation of functional integrity and genomic stability at the cellular level

**Fig. 6:**
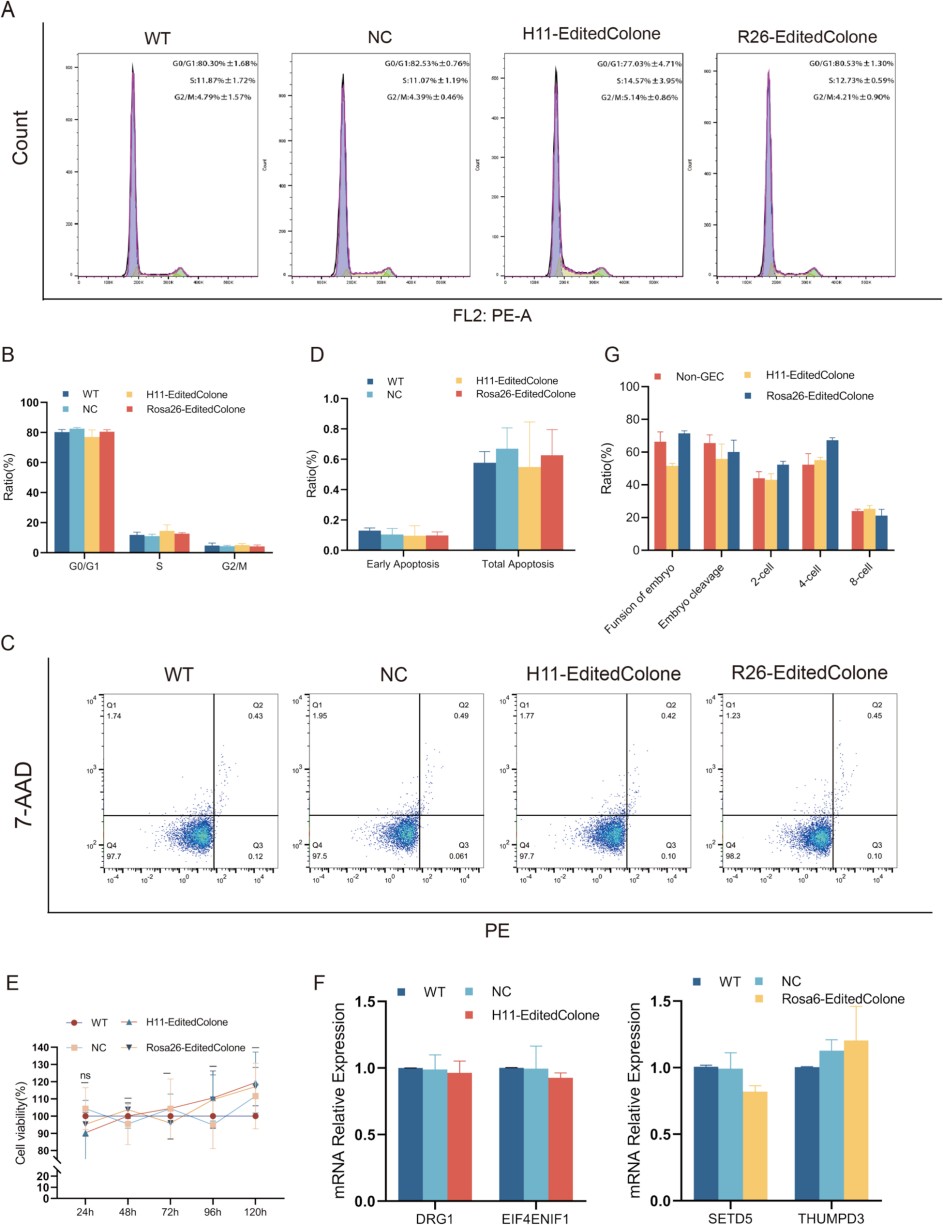
Comprehensive impact of transgene integration on cellular function and embryonic development (Two-way ANOVA with Tukey's post hoc test; Mean ± SD, *n* = 3; NS: *p* > 0.05; GraphPad Prism 8.0). (**A**) Cell cycle distribution analysis. (**B**) Cell cycle phase proportions. (**C**) Apoptosis detection. (**D**) Apoptosis rate. (**E**) Cell proliferation. (**F**) Flanking gene expression. (**G**) Developmental stage statistics of nuclear transfer embryos

To assess the impact of transgene integration on cellular function and the maintenance of genomic stability, we systematically analyzed the key phenotypic features of the gene-edited cell lines (Goat-*H11*-EditedClone series/Goat-*R26*-EditedClone series), along with the expression profiles and genomic characteristics of neighboring genes. Cell cycle analysis revealed no statistically significant differences (*p* > 0.05 by one-way ANOVA for all phases) in the distribution of cells across G0/G1, S, or G2/M phases between the experimental and WT or NC groups (*n* = 3) (Fig. 6A, B). Apoptosis analysis via Annexin-V/PI double staining showed stable early apoptosis rates (*p* > 0.05 by one-way ANOVA) and total apoptosis rates (*p* > 0.05 by one-way ANOVA) across all groups (*n* = 3) (Fig. 6C, D). The CCK-8 proliferation assay further confirmed that there were no significant differences (*p* > 0.05 by one-way ANOVA) in cell viability among the groups after 72 h (Fig. 6E).

RT-qPCR-based evaluation of genomic editing specificity revealed no significant alterations in the transcription levels of genes flanking the target loci. At the *H11* locus, the expression of the adjacent genes *DRG1* (*p* > 0.05 by independent samples t-test) and *EIF4ENIF1* (*p* > 0.05 by independent samples t-test) in the Goat-*H11*-EditedClone series remained unchanged compared with that in WT cells (Fig. 6F). Similarly, at the Rosa26 locus, the transcription levels of *THUMPD3* and *SETD5* in the Goat-*R26*-EditedClone series were not significantly different (*p* > 0.05 by independent samples t-test) from those in WT cells (Fig. 6F). These analyses confirmed the high precision of the genome-editing strategy, with no disruption of transcriptional integrity in the flanking genomic regions.

#### Validation of embryonic developmental compatibility with transgene integration

To evaluate the effects of transgene integration on embryonic development, SCNT was performed using WT or gene-edited (Goat-*H11*-EditedClone series/Goat-*R26*-EditedClone series) donor cells. The results showed no significant differences (*p* > 0.05 by Chi-square test) in the fusion rates, cleavage rates, or developmental progression (2-, 4-, and 8-cell stages) among the WT, Goat-*H11*-EditedClone series, and Goat-*R26*-EditedClone series groups (Fig. 6G, Supplementary Table S12), indicating that transgene integration at the *H11* or *Rosa26* loci did not impair early embryonic development in goats.

#### Longitudinal tracking of growth phenotypes in transgenic cloned goats

Comparative analyses of body weight, length, height, and chest circumference between transgenic (SCNT-WT-Animal 2212, Goat-*R26*-KI-Animal 2270, and Goat-*H11*-KI-Animal 2271) and WT goats at three time points (birth, 6 months, and 12 months) revealed the following trends:

- At birth, the body weights of SCNT-WT-Animal 2212 and Goat-*R26*-KI-Animal 2270 approximated or slightly exceeded the mean WT, whereas #2271 aligned with the lower WT range. The body length and height of #2270 were near or above those of the WT, whereas those of #2271 exhibited significantly lower values.
- At 6 months, the body weight and chest circumference of all transgenic individuals aligned with WT means, whereas body length and height gradually converged toward the WT range.
- At 12 months: All parameters closely matched WT means.

Although the growth trajectories of transgenic individuals broadly paralleled those of the WT, early developmental variations (e.g., elevated body length in #2270 and reduced metrics in #2271 at birth) suggested potential transient fluctuations. However, owing to the limited sample size (n = 1 per genotype), these differences were descriptive and lacked statistical power for hypothesis testing. Future studies with expanded cohorts and rigorous statistical methods are required to clarify potential transgene-related phenotypic effects (Table 2).

**Table 2:** Comparison of body measurements between transgenic and WT goat at different growth stages (*n* = 1 per genotype, data represent preliminary observation)

Timepoint	Group	Body Weight (kg)Mean ± SD	Body Length (cm)Mean ± SD	Height (cm)Mean ± SD	Chest Circumference (cm)Mean ± SD
Birth	2270	3.01	44.2	37.3	38.0
	2271	2.78	41.9	32.8	35.5
	2212	2.94	43.3	36.9	37.1
	Wild-type	2.87 ± 0.14	42.87 ± 1.47	34.67 ± 2.97	36.7 ± 1.35
6months	2270	23.70	57.00	53.90	72.70
	2271	25.20	56.20	57.80	74.20
	2212	23.90	55.70	56.40	74.10
	Wild-type	24.37 ± 0.70	57.33 ± 1.25	56.67 ± 0.47	73.00 ± 1.63
12months	2270	26.00	66.20	60.50	87.10
	2271	26.10	65.70	61.00	87.10
	2212	25.80	67.00	62.10	85.90
	Wild-type	26.17 ± 0.62	67.33 ± 1.25	61.67 ± 0.94	86.67 ± 1.25

## Discussion

This study systematically evaluated the potential of *H11* and *Rosa26* loci as exogenous gene integration targets in goat genome editing. Multi-dimensional validation demonstrated that the *H11* and *Rosa26* loci have high safety profiles, and both loci show stable expression properties, thus providing critical theoretical support for establishing an efficient genome-targeted integration technology in goats (Hu et al. 2021; Huang et al. 2020).

The selection of safe genomic harbor loci remains a core challenge in animal genetic engineering. While previous studies have reported *Rosa26* locus editing in goats (Vats et al. 2021; Li et al. 2024), our study provides the first systematic multi-dimensional evaluation comparing both *H11* and *Rosa26* loci in the context of CRISPR/Cas9-mediated gene editing and somatic cell nuclear transfer. This study revealed that the *H11* locus in goats exhibits unique integration advantages; its localization within a non-coding region of chromosome 17 avoids the risks associated with proximity to essential genes, as seen in traditional loci such as *AAVS1*(Sadelain et al. 2012). Notably, despite interspecies homology below 50%, *H11* retained its cross-species transgene expression efficiency. In contrast, the *Rosa26* locus previously validated in goat embryos (Vats et al. 2021) and more recently using prime editing technology (Li et al. 2024) supported stable transgene expression within its non-coding genomic environment. It is noteworthy that although *Rosa26* is empirically regarded as a safe site in mice, it harbors endogenous non-coding RNA genes and thus does not strictly meet the classical definition of genomic safe harbors (Shrestha et al. 2022). Therefore, is long-term biosafety in large animal models requires independent validation. Experimental data showed a significantly higher integration efficiency at *H11* (67.86%, Fisher Fisher's exact test, *p* < 0.05) than at *Rosa26* (46.15%, Fisher's exact test, *p* < 0.05), which was likely attributable to its lower GC content and more open chromatin conformation (Buenrostro et al. 2015; Schep et al. 2024). This aligns with recent reports on the chromatin state regulation of CRISPR editing efficiency(Schep et al. 2024; Chen et al. 2022), suggesting that future locus screening should integrate multi-dimensional criteria, including sequence features and 3D genomic data (Risca and Greenleaf 2015). Intriguingly, *Rosa26*-mediated *EGFP* expression at the RNA level was 1.86-fold higher than that of *H11*, with a corresponding 25% increase in protein expression, possibly caused by endogenous regulatory elements with broad transcriptional activation properties. This raises a new scientific question: which cis-regulatory elements in the non-coding regions determine the species compatibility of these loci? (Maeso et al. 2016).

Paraffin section fluorescence analysis revealed tissue-specific *EGFP* expression differences between the loci, suggesting distinct regulatory mechanisms. Specifically, *Rosa26* may harbor developmental enhancers, while *H11* achieves cross-tissue stability through an open chromatin architecture (Blayney et al. 2023). These findings provide critical insights for the development of tissue-specific ubiquitous expression systems.

The observed divergence between paraffin section fluorescence microscopy (Fig. 5C) and quantitative image analysis (Fig. S5A) warrants methodological consideration, particularly regarding tissue-specific detection challenges. This discrepancy likely arises from inherent technical characteristics. Direct fluorescence microscopy in paraffin-embedded sections is susceptible to autofluorescence interference, especially in collagen-rich tissues like muscle and dermis where endogenous fluorophores (e.g., collagen/elastin emitting at 450–550 nm) can mimic EGFP signals, potentially generating false positives as seen in the muscle tissue of Goat-*R26*-KI-Animal 2270 (Engel et al. 2006). Conversely, threshold-based quantification approaches may underestimate true expression levels when conservative settings exclude authentic signals in heterogenous tissues, such as dispersed *EGFP*^+^–cells in muscle. To enhance detection reliability in future studies, we recommend implementing cryosectioning to minimize autofluorescence artifacts, validating findings through anti-*GFP* immunohistochemistry, and employing spectral unmixing techniques to differentiate specific *EGFP* signals from background fluorescence in complex mammalian tissues (Xu et al. 2021).

CRISPR/Cas9 optimization faces the dual challenges of off-target effects and repair efficiency. Off-target activity occurs when Cas9 cleaves genomic regions with sequence similarities to sgRNA (Veres et al. 2014; Dong et al. 2022; Hiranniramol et al. 2020). Strategies to enhance specificity include modifying Cas9 nuclease activity (Kleinstiver et al. 2016; Kocak et al. 2019), optimizing sgRNA design (Chuai et al. 2018; Doench et al. 2016; Zhang et al. 2023a, b), and using bioinformatics tools to minimize off-target risks. This study improved the HDR efficiency to practical levels through sgRNA optimization and RS-1 co-treatment (Jeon et al. 2020; Aoshima et al. 2021; Zhao et al. 2024). Current HDR enhancement methods include cell cycle synchronization, NHEJ pathway inhibition (e.g., SCR7) (Maruyama et al. 2015; Anuchina et al. 2023), and HDR activators such as RS-1.

Multi-dimensional safety assessments confirmed that *H11*/*Rosa26* integration had no significant effects on cellular function or embryonic development, thus providing critical guidance for transgenic animal production. However, current evaluations have focused on the transcriptomic and proteomic levels; potential epigenetic effects (e.g., DNA methylation ripple effects) require multi-omics analyses (Přibylová et al. 2022). Therefore, long-term monitoring of cross-generational genetic risks in livestock is essential.

Donor cell aging, which is linked to epigenetic barriers in nuclear reprogramming (Zhang et al. 2023a, b; Xu et al. 2023; Loi et al. 2016), limits SCNT efficiency. Maintaining subconfluent passaging of monoclonal cells preserves viability, potentially by preventing the accumulation of repressive histone modifications (e.g., *H3K27me3*) and silencing cell cycle inhibitors (e.g., *CDKN1A*). Optimal cryopreservation density mitigates age-related issues (Glanzner et al. 2018).

While this study validated the applicability of *H11* and *Rosa26* loci, several key challenges persist. The comparatively lower integration efficiency at Rosa26 (46.15% vs. H11's 67.86%, *p* < 0.05) necessitates strategic improvements such as homology arm optimization or epigenetic modulation (Baker et al. 2017; Spicuglia et al. 2010). Concurrently, long-term culture of gene-edited cells revealed proliferative slowdowns, coupled with unresolved risks of epigenetic silencing during cell passaging (Chen et al. 2023). Critically, the limited transgenic cohort size (*n* = 2 viable offspring) and low birth rates fundamentally constrain phenotypic analysis. To address these challenges, future research should prioritize developing tissue-specific expression systems to refine Rosa26's ubiquitous activity (Li et al. 2019); establishing multi-generational stability tracking in transgenic lineages; and exploring novel gene delivery systems for large DNA fragment transfection (Miyazaki and van der Meer 2013).

This work provides foundational support for safe genome editing in goats, offering a technical paradigm for livestock genetic improvement. However, phenotypic observations derived from only two transgenic individuals require cohort expansion to exclude stochastic effects. Further exploration of chromatin 3D structure-locus efficiency relationships will advance precision editing in domestic animals (Huang and Wu 2016).Importantly, this work presents a proof-of-concept platform. Translating this into practical applications will require optimization of donor cell reprogramming protocols to improve nuclear transfer efficiency and scaling up recipient ewe numbers to increase transgenic offspring yield. These advancements will enable statistically powered biosafety assessments, particularly for long-term monitoring of genomic instability and epigenetic alterations.

## Conclusions

This study successfully established a cashmere goat model with *EGFP*-targeted integration at the *H11* and *Rosa26* loci using CRISPR/Cas9 gene editing combined with somatic cell nuclear transplantation. The key conclusions are as follows:

1. Integration Site Identification: For the first time, The *H11* locus was identified as a safe harbor site and *Rosa26* as a widely applicable targeting platform on goat chromosomes 17 and 22.
2. High-Efficiency Expression Validation: Both loci supported stable *EGFP* expression across developmental stages.
3. Developmental Safety: Targeted integration did not disrupt cellular function, embryonic development, or individual growth, thus providing a reliable technical platform for genetic improvement in cashmere goats.

## Supplementary Information

Below is the link to the electronic supplementary material.

Supplementary file1 (DOCX 129925 KB)

## Data Availability

No datasets were generated or analysed during the current study.
