# Supplementary material for: Validation of caprine H11 and the Rosa26 platform for transgene integration via CRISPR-based system: investigations on stable transgene expression and genetic biosafety
Source: Funct Integr Genomics. 2025 Aug 29;25(1):180. doi: 10.1007/s10142-025-01679-x (PMC12397189; doi:10.1007/s10142-025-01679-x)
Supplement: Supplementary file 1 — Supplementary file1 (DOCX 129925 KB) [file 10142_2025_1679_MOESM1_ESM.docx]

Table S1 Primer sequences for RT-qPCR analysis

| Gene symbol | Primer Direction | Primer Sequence (5'→3') | Amplicon Length(bp) | Annealing Temp (℃) |
| --- | --- | --- | --- | --- |
| Rosa26 | Forward | CAGATACCGGCCAGACGAC | 144 | 62．0 |
|  | Reverse | CGGGGCCACCTAGAGAAGA |  |  |
| EGFP | Forward | AGATCCGCCACAACATCGAG | 206 | 60.0 |
|  | Reverse | GTCCATGCCGAGAGTGATCC |  |  |
| DRG1 | Forward | GCTCGGACTCAGAAGAACA | 182 | 56.0 |
|  | Reverse | CCCACAGACGGAAAACC |  |  |
| EIF4ENIF1 | Forward | ACGAACAGCCTCTGTGAAGG | 196 | 61.0 |
|  | Reverse | AGCCAAGCATGGCACCTTAT |  |  |
| SETD5 | Forward | CTGGAGTACCGCAAACG | 201 | 54.0 |
|  | Reverse | GGAATGAGACGGGGAGA |  |  |
| THUMPD3 | Forward | ACGAAAATCCAACCATCA | 228 | 53.0 |
|  | Reverse | TCATATCAGCCTTCCACTTA |  |  |
| GAPDH | Forward | GGTCGGAGTGAACGGAT | 174 | 54.0 |
|  | Reverse | TCTGCCTTGACTGTGCC |  |  |

Table S2 CRISPR/Cas9 gene editing primers and amplification parameters

| Target Locus | Function Category | Primer Name | Sequence (5'→3') | Length  (bp) | Annealing Temp (℃) |
| --- | --- | --- | --- | --- | --- |
| H11 | sgRNA Efficacy Screening | H11_sgEff_sg1_F | CCCCTGTTGCTTGAGACTGT | 745 | 61.5 |
|  |  | H11_sgEff_sg1_R | TTTGTCATGTTTACTGGGGCAA |  |  |
| H11 | sgRNA Efficacy Screening | H11_sgEff_sg2_F | TCCCCACTTTACACCCTGGA | 517 | 62.5 |
|  |  | H11_sgEff_sg2_R | TCAGCCAGTCTCTCAGCTCT |  |  |
| Rosa26 | sgRNA Efficacy Screening | R26_sgEff_sg1_F | ACGCAGTCTCGCTGCATAAA | 749 | 59.0 |
|  |  | R26_sgEff_sg1_R | GGGAACGGCCCCTTGTTATT |  |  |
| Rosa26 | sgRNA Efficacy Screening | R26_sgEff_sg2_F | GACAAGACTAGCCCGACCTG | 689 | 63.0 |
|  |  | R26_sgEff_sg2_R | TCTGTTTTGGCGGTGTAGCA |  |  |
| H11 | Homology Arm Left | H11_HAL_F | GAGACATGTAGACCTGGCTGTACTTCCCC | 993 | 68.0 |
|  |  | H11_HAL_R | GCTGGATTAATGGTCGCTTTTCAACAAAACCTG |  |  |
| H11 | Homology Arm Right | H11_HAR_F | GCAACGCGTAGAGCTGAGAGACTGGCTGA | 1158 | 69.5 |
|  |  | H11_HAR_R | GCAACGCGTGAGTACCGACAGTGAGCAGG |  |  |
| Rosa26 | Homology Arm Left | R26_HAL_F | GAACATGTGACGGGAGCATAGCGTTTGT | 854 | 67.0 |
|  |  | R26_HAL_R | TGTGTTATTAATCTCGCGCCGATTTTTGGAGA |  |  |
| Rosa26 | Homology Arm Right | R26_HAR_F | CCCTCACGCGTCCCAATTTCTTTATCTTCCC | 916 | 70.0 |
|  |  | R26_HAR_R | CCTACGCGTTCCTGTCAGTAGTTACCACCC |  |  |
| H11 | Knock-in Validation | H11-KI-F | TCCCCACTTTACACCCTGGA | 712/2157 | 63.5 |
|  |  | H11-KI-R | CCTCAAGACTCTGGTGCCTG |  |  |
| Rosa26 | Knock-in Validation | R26-KI-F | TCTGTTTTGGCGGTGTAGCA | 700/2056 | 62.0℃ |
|  |  | R26-KI-R | GCTGCCTGAAGGACAAGACT |  |  |

Table S3 Validated sgRNA spacer sequences for targeting the caprine

*H11* and *Rosa26* safe harbor loci

| sgRNA ID | Spacer Sequence (5' to 3') | Required PAM (3') |
| --- | --- | --- |
| gRNA-H11-1 | CGGGGTGCAGCCTATTAACC | TGG |
| gRNA-H11-2 | GTGGGCAGCTCCAGGTTAAT | AGG |
| gRNA-R26-1 | GTCGAGTCTCTCCTCGATTA | TGG |
| gRNA-R26-2 | GAGGCGATGACGAGATCGCG | GGG |

Table S4 Electroporation parameters for plasmid DNA transfection of fibroblast cells

| Voltage (V) | Pulse Width (ms) | Cell Number | DNA Amount (µg) |
| --- | --- | --- | --- |
| 175 | 7.5 | 1×10^6^ | 20.0 |
| 200 | 5.0 | 1×10^6^ | 20.0 |
| 225 | 5.0 | 1×10^6^ | 20.0 |
| 250 | 2.5 | 1×10^6^ | 20.0 |

Table S5 Predicted off-target sites

Table S6 Primers for detection of potential off-target editing at the caprine

*H11* locus and predicted off-target sites.

| Genomic Locus | Primer name | Sequence (5’-3’) | Length (bp) | Annealing Temp (℃) |
| --- | --- | --- | --- | --- |
| Chr7-1：NC_030814.1:  33893835-33893857 | Chr7-1-F | TACTCTATACTATATGTGCTGCA | 311 | 62.0 |
|  | Chr7-1-R | GTGTTAGGGTGAAGGGCATAA |  |  |
| Chr7-2：NC_030814.1:  108272163-108272185 | Chr7-2-F | CAATGTTATGTGGCAGTC | 254 | 51.0 |
|  | Chr7-2-R | TTCCAGGTATTTGAAGGT |  |  |
| Chr15：NC_030822.1:  42881558-42881580 | Chr15-F | AAAACACTCCCACGTTGTGC | 355 | 62.0 |
|  | Chr15-R | ATGGAAATGGGAGAACCACCG |  |  |
| Chr18：NC_030825.1  9169406-9169428 | Chr18-F | CCTCCCAAAGATAGGCACA | 391 | 57.0 |
|  | Chr18-R | AACCAGCAAGGACCCACT |  |  |
| Chr20：NC_030827.1:  36956344-36956366 | Chr20-F | TTCAACCAGTCCACCTCTACA | 644 | 63.0 |
|  | Chr20-R | AAGATGTTGCGGGGAAATGACA |  |  |

Table S7 Primers for detection of potential off-target editing at the caprine

*Rosa26* locus and predicted off-target sites

| Genomic Locus | Primer name | Sequence (5’-3’) | Length (bp) | Annealing  Temp (℃) |
| --- | --- | --- | --- | --- |
| Chr2：NC_030809.1:  22159187-22159209 | Chr2-F | AGCTGTTTGGGACTGCTCTC | 387 | 59.0 |
|  | Chr2-R | ACCCCAAACCTGCTGTGAAA |  |  |
| Chr16：NC_030823.1:  75835081-75835103 | Chr16-F | GAGAGGGACCCCCATCTTCT | 600 | 65.0 |
|  | Chr16-R | AAGGACAAGCACAGAGACCG |  |  |
| Chr26：NC_030833.1:  7859711-7859733 | Chr26-F | CATTTCGGCAGGCAACTCAG | 556 | 59.0 |
|  | Chr26-R | TTTCCGGTTGAAATTGCGGG |  |  |
| Chr28：NC_030835.1:  29593283-29593305 | Chr28-F | ACACTCAGACTAGAATTGTGAAGC | 601 | 68.0 |
|  | Chr28-R | AGGAGAAAAGCACTCTCAAGCA |  |  |
| Chr15：NC_030822.1:  1293585-1293607 | Chr15-F | TTGTTGCCGTTGTTCAGCAG | 616 | 58.0 |
|  | Chr15-R | TAGTCCAAACGAGCAGCCAG |  |  |

Table S8 Composition of the collection medium for COCs

| Reagent Name | Vendor | Catalog Number | Amount Added |
| --- | --- | --- | --- |
| M199 | Gibco, USA | 31100-035 | 9.60 g |
| Hepes | Sigma, Germany | H4034 | 1.19 g |
| NaHCO₃ powder | Wako, Japan | 191-01305 | 2.10 g |
| Penicillin-Streptomycin (PS) | VivaCell, China | C3420 | 10.00 μL |
| Fetal Bovine Serum **(**FBS) | VivaCell, China | C04001 | 20.00 mL |
| Heparin | Wako, Japan | 081-00136 | 1.00 mL |
| Embryo-Tested Water | Sigma, Germany | W1503 | up to 1000.00 mL |

‡ The pH (7.2–7.4) and osmolarity (280–300 mOsm/kg) of the complete medium were measured at 37°C.

Table S9 Composition of the in vitro maturation medium for COCs

| Reagent Name | Vendor | Catalog Number | Amount Added |
| --- | --- | --- | --- |
| M199 | Gibco, USA | 12340-030 | 18.00 mL |
| **Caprine Estrus Serum** (CES) | / | / | 2.00 mL |
| Estradiol(1000×) | Wako, Japan | 058-04043 | 20.00 μL |
| Luteinizing Hormone (LH) | NSHF, China | / | 20.00 μL |
| Follicle-Stimulating Hormone (FSH) | NSHF, China | / | 20.00 μL |
| Penicillin-Streptomycin (PS) | VivaCell, China | C3420 | 200.00 μL |
| Sodium pyruvate | Wako, Japan | 199-03062 | 0.0044 g |
| HEPES | Sigma, Germany | H4034 | 0.0476 g |
| Embryo-Tested Water | Sigma, Germany | W1503 | up to 20.00 mL |

‡ The pH (7.2–7.4) and osmolarity (280–300 mOsm/kg) of the complete medium were measured at 37°C.

Table S10 Composition of the embryo development medium

| Reagent Name | Vendor | Catalog Number | Amount Added |
| --- | --- | --- | --- |
| SOF solution | / | / | 19.20 mL |
| L-Glutamine | Sigma, Germany | G8540 | 20.00 μL |
| Myo-inositol | Wako, Japan | 094-00281 | 10.00 mg |
| Essential Amino Acids (EAA) | Sigma, Germany | B6766 | 600.00 μL |
| Non-Essential Amino Acids (non-EAA) | Sigma, Germany | M7145 | 200.00 μL |
| Bovine Serum Albumin (BSA) | Sigma, Germany | A6003 | 0.12 g |
| Embryo-Tested Water | Sigma, Germany | W1503 | up to 20.00 mL |

‡ The pH (7.2–7.4) and osmolarity (280–300 mOsm/kg) of the complete medium were measured at 37°C.

Table S11 Genotype distribution of cleavage-stage reconstructed embryos with site-specific modifications

| Genotype | Number of embryos |
| --- | --- |
| Wild-Type | 44 |
| H11 locus-Edited | 249 |
| Rosa26 locus-Edited | 205 |
| Total | 498 |

Percentages calculated based on total cleavage-stage reconstructed embryos (n=498)

Table S12 Key indicators of embryonic development

| Developmental indicator | WT | Goat-H11-EditedClone | Goat-Rosa26-EditedClone |
| --- | --- | --- | --- |
| Fusion rate (%) | 66.33 ± 6.04 | 51.64 ± 1.41 | 71.53 ± 1.54 |
| Cleavage rate (%) | 65.58 ± 4.97 | 55.86 ± 9.14 | 60.15 ± 7.15 |
| 2-cell rate (%) | 44.06 ± 3.94 | 43.10 ± 3.70 | 52.35 ± 2.01 |
| 4-cell rate (%) | 52.34 ± 6.67 | 55.12 ± 1.68 | 67.28 ± 1.50 |
| 8-cell rate (%) | 24.03 ± 1.08 | 25.37 ± 2.04 | 21.24 ± 3.82 |

Figure S1. Genomic structure and homology analysis of caprine H11 locus and Rosa26 gene

A. Schematic diagram of H11 locus genomic localization (highlighted by blue dashed ellipse)

B. Bioinformatics prediction of caprine H11 locus and flanking sequences

C. Homology alignment of H11 gene and its flanking sequences

D. Genomic localization of Rosa26 gene across multiple species (Ensembl database; indicated by red arrows)

E. Comparative analysis of caprine genome with mouse Rosa26 promoter and exon 1 based on Ensembl database

F. Comparative analysis of caprine genome with mouse Rosa26 promoter and exon 1 based on NCBI database (accession number: NC_000072.6).The red line indicates the first exon sequence of the caprine Rosa26 gene.

G. Sequence alignment analysis of Rosa26 gene between ovine and caprine

H. Homology comparison of Rosa26 first exon among multiple species

Figure S2. Construction of the Gene Editing System and Optimization of Electrotransfection Conditions

(A) Sanger sequencing validation of sgRNA target sites (yellow highlight: sgRNA complementary region).

(B) Schematic structure of the Cas9/sgRNA co-expression vector.

(C) Schematic of the H11-EGFP gene-targeting vector (generated with SnapGene v5.0).

(D) Schematic of the Rosa26-EGFP gene-targeting vector (generated with SnapGene v5.0).

(E) Screening of electroporation conditions (effects of voltage and pulse duration on transfection efficiency). Scale bar =100μm.

Fig. S3 Sequencing alignment of potential off-target sites for H11 sgRNA

(A-E) Sequencing results for five potential off-target loci (Chr7-1, Chr7-2, Chr15, Chr18, Chr20). Green lines indicate the location of validation primers. Red regions highlight genomic sequences at the sgRNA off-target sites.

Fig. S4 Sequencing alignment of potential off-target sites for Rosa26 sgRNA

(A-E) Sequencing results for five potential off-target loci (Chr2, Chr16, Chr26, Chr28, Chr15). Green lines indicate the location of validation primers. Red regions highlight genomic sequences at the sgRNA off-target sites.

Fig. S5 Tissue-Specific Fluorescence Expression: Cross-Individual Comparisons and Intra-Individual Hetero-geneity (Note: Fluorescence intensity normalized to sample in row 1)

(A) Cross-individual analysis: Fluorescence intensity in matched tissues of animals #2212, #2270, and #2271. Error bars: mean ± SD. *p < 0.05; **p < 0.01; ***p < 0.001; ****p < 0.0001.

(B) Intra-individual heterogeneity: Tissue-specific expression patterns in animals #2270 and #2271. Error bars: mean ± SD. *p < 0.05; **p < 0.01; ***p < 0.001; ****p < 0.0001.

Fig. S6: WT-controlled genotyping confirms successful editing in H11 (712bp/2157bp) and Rosa26 (700bp/2056bp) cell lines
